# Utility of clinical, laboratory, and lymph node MYD88 *L265P* mutation in risk assessment of diffuse large B-cell lymphoma patients

**DOI:** 10.1186/s43046-024-00237-z

**Published:** 2024-10-14

**Authors:** Ahmed Talaat Hanbal, Shaimaa El-Ashwah, Ahmed E. Eladl, Sameh Shamaa, Layla M. Saleh

**Affiliations:** 1 Clinical Hematology, Internal Medicine Department, Oncology Center, Mansoura University, PO Box 35516, Mansoura, Egypt; 2 Hematology Section, Clinical Pathology Department, Faculty of Medicine, Mansoura University, PO Box 35516, Mansoura, Egypt; 3 Pathology Department, Faculty of Medicine, Mansoura University, PO Box 35516, Mansoura, Egypt; 4 Medical Oncology and Internal Medicine, Oncology Center, Mansoura University, PO Box 35516, Mansoura, Egypt

**Keywords:** DLBCL, MYD88, OS, DFS

## Abstract

**Background:**

Diffuse large B-cell lymphoma (DLBCL) is an aggressive non-Hodgkin lymphoma and is characterized by heterogeneity in biology and clinical behavior. Mutations in the myeloid differentiation primary response 88 (MYD88) are found in different lymphoproliferative disorders and are associated with variable clinical and prognostic impact.

**Aim:**

To investigate the frequency of *MYD88 L265P* mutation and its clinical impact in a cohort of Egyptian DLBCL patients.

**Methods:**

FFPE lymph node samples from 87 DLBCL patients (46 males / 41 females; median age, 58 years) were included and analyzed for MYD88 L265P by an allele-specific PCR.

**Results:**

*MYD88* L265P mutations were found in 52 patients (59.8%) out of 87 DLBCL cases. Patients with L265 mutation were significantly younger than non-mutated patients (*p* = 0.022). None of the patients with the L265P mutation showed a significant association with the clinical parameters of DLBCL. Interestingly, MYD88 L265 mutated patients were found to be significantly correlated with HCV infection (*p* = 0.037). The median follow-up time across the entire cohort was 26 months. Univariate analysis showed that overall survival (OS) was affected by gender, LDH level, and CNS-IPI scoring (*p* = 0.048, 0.008, and 0.046, respectively), while disease-free survival (DFS) was affected by B symptoms and LDH level (*p* =  < 0.000 and 0.02, respectively). However, the MYD88 mutation status and other prognostic factors showed no association with OS or DFS.

**Conclusions:**

Our findings indicate a high frequency of *MYD88* L265P mutations in our study population and not associated with prognostic markers or the outcome of the disease.

## Introduction

Diffuse large B-cell lymphoma (DLBCL) is a heterogeneous disease regarding morphology, immunophenotyping, genetic aberrations, as well as clinical presentation and patient outcome [1, 2]. It accounts for about 25% of all non-Hodgkin’s lymphoma (NHL) patients worldwide [3]. Interestingly, NHL was reported as the 5th cause of cancer mortality in Egypt [4].

Subtypes of DLBCL related to the different cell of origin (COO) have been recognized according to gene expression profiling (GEP) into the following: germinal center B-cell like (GCB), activated B-cell like (ABC), and primary mediastinal B-cell lymphoma (PMBL) [5]. Each subtype is found to be associated with distinct genetic lesions and oncogenic pathways involved in tumor development [6].

GEP studies have shown unfavorable prognosis of ABC DLBCL subtype depending on the activation of NF-κB transcription complex blocking apoptosis, leading to tumor cell survival and treatment resistance [7]. A variety of signaling pathways can induce NF-κB transcription complex, including B-cell receptor (BCR), CD40, and toll-like receptor (TLR) [8].

Myeloid differentiation primary response 88 (MYD88) is an activated protein in the early genetic responses of myeloid cells to differentiation and growth inhibitory stimuli, in addition to its central role in immunity [9].

MYD88 acts as an adaptor protein that activates the NF-κB signaling through toll-like (TLRs) and interleukin-1 receptors. Upon TLR activation, MYD88 is phosphorylated and subsequently recruits IL-1R-associated kinases (IRAKs) and many other downstream proteins resulting in NF-κB, JAK kinase / STAT3 activation and secretion of IL6, IL10, and interferon-β [10].

It was recently discovered that a leucine (CTG) to proline (CCG) substitution at position 265 (L265P) in the coding region of the MYD88 occurred in 29% of ABC DLBCL, and has the most severe oncogenic effect in this pathway [11]. So, MYD88 provides an attractive target especially in patients with DLBCL who do not respond well to the anti-CD20 antibody (rituximab). Thus, *MYD88* L265P mutation status might be a good predictor of response to chemotherapy in DLBCL patients [10].

The *MYD88* L265P mutation occurs at various frequencies in DLBCL [12, 13] and being related to specific extranodal sites, including 69% of primary cutaneous leg type DLBCL, 38–50% of central nervous system lymphomas and 9% of MALT lymphomas [14, 15]. It has been also reported to be associated with unfavorable outcome [16].

Moreover, the prognostic value and clinical impact of the *MYD88* L265P mutation have been a matter of controversy among different lymphoproliferative disorders [10]. However, few or even no studies have been published investigating *MYD88* L265P mutations in Egyptian patients with DLBCL.

Therefore, in this study, we aimed to investigate the frequency of *MYD88* L265P mutation in a series of patients with de novo DLBCL attending Oncology Center Mansoura University (OCMU). Moreover, we aimed to determine the importance of different clinical laboratory in addition to lymph node *MYD88* L265P mutation in risk assessment of DLBCL patients.

## Materials and methods

### Patient sampling and characteristics

The current study was approved by the institutional review board of the Faculty of Medicine, Mansoura University, Egypt. Written informed consent was obtained from all participants in accordance with the Declaration of Helsinki.

This retrospective study was conducted on a total of 87 patients (46 males / 41 females; median age, 58 years), diagnosed with DLBCL according to WHO classification [17]. All samples were diagnosed and selected by an expert hematopathologist from the time period of 2016 to 2019. Availability of the histological material was a major inclusion criterion. Patients with follicular lymphoma or any other type of indolent lymphoma with subsequent transformation into DLBCL were excluded. Each patient received an anthracycline containing regimen, with or without rituximab. The end point of clinical follow-up was either the date of the last contact or the date of death.

All patients were classified according to the Ann Arbor staging system and International Prognostic Index (IPI) score, following the criteria described in previous studies [18, 19].

Blood cell counts and serum biochemistry including lactate dehydrogenase (LDH) were assessed. HCV seropositivity was detected by electrochemiluminescence (ECL) technique using Cobas e-411 analyzer (Roche Diagnostics, Germany). Chest, abdomen, and pelvis computerized tomography scans (PET/CT), in addition to bone marrow biopsy and response to therapy, were assessed according to conventional criteria [20].

### Histopathologic characterization

In most samples, immunohistochemistry was performed for CD20, CD10, BCL6, MUM1, and BCL2. The Hans’ algorithm [21] was used for the COO classification, in addition to Ki67 to determine the proliferation cell index. Formalin-fixed and paraffin-embedded (FFPE) tissue samples were obtained during standard diagnostic procedures.

### DNA extraction and AS-PCR assay for MYD88 L265P

DNA was extracted from 5- to 10-µm-thick formalin-fixed paraffin-embedded (FFPE) sections of DLBCL lymph node (LN) samples with QIAamp DNA FFPE Kit (QIAGEN GmbH, Germany) following the manufacturer instructions. Briefly, we used 50 ng of extracted DNA to amplify with each of the forward and reverse primers for wild and mutant alleles. The mutant-specific reverse primer was 5′-CCT TGT ACT TGA TGG GGA aCG-3′, and the wild-type-specific reverse primer was 5′-GCC TTG TAC TTG ATG GGG AaC A-3′. The common forward primer was 5′-AAT GTG TGC CAG GGG TAC TTA G-3′. PCR reaction was performed in a final volume of 25 µL with 50 nM of each primer and 50 ng DNA using COSMO PCR RED Master Mix (Willowfort, Birmingham, UK). Thermal cycling conditions were as follows: 2 min at 94 °C, followed by 40 cycles of 94 °C for 30 s, 57 °C for 30 s, and 68 °C for 30 s, with a final extension at 68 °C for 5 min. The amplified PCR products (159 bp) were separated on 2% agarose gel as previously described [22].

### Statistical analysis

Data were analyzed with SPSS version 22. The normality of data was first tested with a one-sample Kolmogorov–Smirnov test. The association between categorical variables was tested using the chi-square test and the Fischer exact test. Continuous variables were presented as mean ± SD (standard deviation) for parametric data and median for non-parametric data. Patient survival data were analyzed using the Kaplan–Meier method. Differences in survival were tested by the log-rank test. Overall survival (OS) was calculated from the date of diagnosis to the date of death or the last date of follow-up. Progression-free survival (PFS) was calculated from the date of diagnosis to the first date of disease progression, relapse, or death as a result of any cause or the last date of follow-up. The results were considered statistically significant if *p* < 0.05.

## Results

### Patient characteristics

In this study, 87 patients were included, 46 males (52.9%) and 41 females (47.1%). The median age at diagnosis was 58 years (range 46–65 years). Of the 87 patients, 7 (8%) were extranodal, 52 (60%) were nodal and 28 (32%) were both nodal and extranodal lymphoma, and 28 (32%) patients presented with B symptoms. At the time of diagnosis, 66 (75.8%) patients had elevated serum lactate dehydrogenase (LDH), 40 (46.8%) of patients were HCV positive, and 37 (42.5%) scored a high International Prognostic Index (IPI). Twenty-four patients (27.6%) were in the early stage (I and II) and 63 (72.4%) with the late stage (III and IV). GCB and non-GCB subtypes were 34 (43%) and 45 (57%) patients, respectively. Seventy-nine patients received chemotherapy, and 62 (78.5%) of them received CHOP ± R. Rituximab involved only in 24% of treatment regimens. Evaluation for response was assessed in 74 patients only, who completed chemotherapy, 41(47.1%) of them achieved complete response, 10 (11.5%) partial response, 12 (13.8%) showed stable disease, and 11 (12.6%) had progressive disease, while 13 (15%) patients did not undergo evaluation. Relapse was observed in only 6 out of 41 assessed patients (14.6%) during their clinical courses, and 22 (25.3%) died of lymphoma. The median follow-up of the patient survival was 26 months. Main variables were recorded and analyzed according to MYD88 mutational status recorded in Table 1, in addition to the main characteristics of the studied patients.

**Table 1:** Clinicopathological characteristics of DLBCL patients

	**Number**	**%**	**MYD88 L265P mutation**		***P* value**
	**Total = 87**		**Negative (*n* = 35) (40.2%)**	**Positive (*n* = 52) (59.8%)**	
**Gender**					0.9
** Male**	46	53%	19	27	
** Female**	41	47%	16	25	
**Age (median-range)**	58 (46–65)		60 (54–65)	52.5 (39–65)	***0.022***
** ≥ 60 years**	38	44	19	19	0.125
** < 60 years**	49	56	16	33	
**Performance status**					0.051
** < 2**	66	76	23	43	
** ≥ 2**	21	24	12	9	
**Anatomical sites**					0.104
** Nodal**	52	60	17	35	
** Extranodal**	7	8	5	2	
** Nodal+extranodal**	28	32	13	15	
**B symptoms**					0.816
** Absent**	59	68	23	36	
** Present**	28	32	12	16	
**Splenic involvement (*n* = 84)**					0.369
** Normal**	48	57	17	31	
** Enlarged**	36	43	17	19	
**IPI score**					0.454
** Low**	23	26	6	17	
** Low-intermediate**	27	31	12	15	
** High intermediate**	24	28	11	13	
** High**	13	15	6	7	
**CNS-IPI**					0.266
** Low**	23	26.5	6	17	
** Intermediate**	50	57.5	23	27	
** High**	14	16	6	8	
**LDH**					0.802
** Normal**	21	24	9	12	
** High**	66	76	26	40	
**Subgroup (*n* = 79)**					0.643
** GCB**	34	43	12	22	
** Non-GCB**	45	57	19	26	
**Stage**					0.471
** Low (I–II)**	24	28	8	16	
** High (III–IV)**	63	72	27	36	
** BCL2 (*n* = 38)**					0.69
** Positive**	28	26	9	19	
** Negative**	10	74	2	8	
**CD10 (*n* = 81)**					0.146
** Positive**	26	32	7	19	
** Negative**	55	68	25	30	
**BCL6 (*n* = 76)**					0.99
** Positive**	53	70	22	31	
** Negative**	23	30	10	13	
**MUM1 (*n* = 70)**					
** Positive**	43	61	16	27	0.456
** Negative**	27	39	13	14	
**Ki67 labeling index (*n* = 29)**					
** ≥ 60%**	21	72	6	15	0.39
** < 60%**	8	28	4	4	
**HCV (*n* = 86)**					***0.037***
** Positive**	40	47	21	19	
** Negative**	46	53	14	32	
**Treatment protocol (*n* = 79)**					0.287
** Chemotherapy**	60	76	20	40	
** Rituximab/chemotherapy**	19	24	9	10	
**Response to treatment (*n* = 74)**					0.872
** PR **	10	14	3	7	
** PD**	11	15	3	8	
** SD**	12	16	4	8	
** CR**	41	55	16	25	
**ORR (*n* = 74)**					0.61
**Yes**	51	69	19	32	
**No**	23	31	7	16	
**Relapse (*n* = 42)**					0.99
** Yes**	6	14	2	4	
** No**	36	86	14	21	
**BM infiltration (*n* = 85)**					0.99
** Yes**	8	9	3	5	
** No**	77	91	31	46	
**Survival months (median, range)**	16 (7.6–24)		17.5 (4.8–25.3)	15.5 (9–24)	0.871
**Patient status**					0.41
** Dead **	22	25	9	13	
** Still alive**	46	53	16	30	
** Lost follow-up**	19	22	10	9	

Bolded, italicized values represent significant *P* values < 0.05

### Correlation between MYD88 L265P status and clinical characteristics

**Fig. 1:**
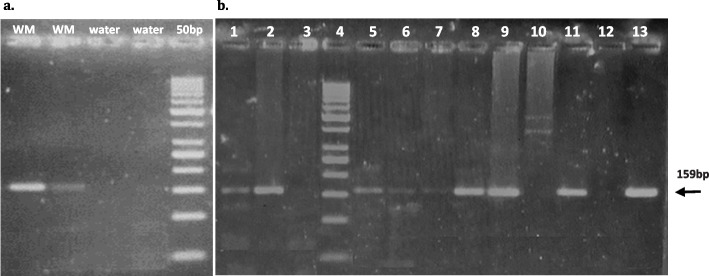
Allele-specific polymerase chain reaction (AS-PCR) of the MYD88 L265 mutation in DLBCL patients. Ten microliters of PCR products were separated by electrophoresis through 2% agarose gel, stained with ethidium bromide, and visualized by ultraviolet illumination. The size of the products is indicated on the right. **a** Waldenström’s macroglobulinemia patient samples used as positive control (WM) bands detected in both lanes. Molecular grade water used as negative control (no bands) and 50 bp ladder. **b** DLBCL patients. Bands were detected in samples with L265P mutation in lane 1, lane 2, lane 5, lane 6, lane 7, lane 8, lane 9, lane 11, and lane 13. No bands detected in lane 3, lane 10, and lane 12, 50 bp ladder in lane 4

MYD88 L265P mutation was observed in 52 (59.8%) of 87 DLBCL cases (Fig. 1). Patients with the L265P mutation were 27 males and 25 females, with a median age of 52.5 years, ranging from 39 to 65 years. Nineteen patients were older than 60 years. Patients with L265 mutation were significantly younger than non-mutated patients (*p* = 0.022). None of the patients with the L265P mutation showed a significant association with clinical parameters of DLBCL, including patient’s gender, tumor location, B symptoms, performance status, LDH level, IPI score, immunohistochemical subtype, clinical stage, and splenic involvement.

Interestingly, MYD88 L265-mutated patients were found to be significantly correlated with HCV infection (*p* = 0.037), 32 HCV-negative patients in the mutated group versus 14 in thr non-mutated group. Also, MYD88 L265 patients showed significantly higher platelet count in comparison to the non-mutated group (*p* = 0.026) (Table 2) as many HCV-negative patients belong to the mutated group. However, no significant association of the MYD88 L265 mutation was observed with other laboratory parameters.

**Table 2:** Laboratory characteristics of DLBCL patients

	**DLBCL patients (median, range) (*n* = 87)**	**MYD88 L265P mutation**		*P* value
		**Negative (*n* = 35)**	**Positive (*n* = 52)**	
**WBCs × 10**^**3**^**/µl**	8 (5.5–10.5)	8 (5.7–11.7)	7.8 (5.4–9.5)	0.436
**ALC × 10**^**3**^**/µl**	1.7 (1.1–2.5)	1.8 (1.3–2.5)	1.7 (0.8–2.3)	0.283
**Platelet × 10**^**3**^**/µl**	231 (185–305)	203 (145–258)	238 (207–329)	***0.026***
**Hemoglobin level (g/dl) **	11.6 (10.4–13.3)	11.6 (10.5–12.9)	12.1 (10.3–13.4)	0.88
**ESR 1st hour**	30 (15–50)	35 (20–52.5)	23.5 (11.8–40)	0.123
**LDH (U/L)**	331 (255–449)	402 (279–524)	322 (253–412)	0.311
**ALT (IU/L)**	22 (15–35)	23 (16–33)	22 (13.3–40)	0.655
**AST (IU/L)**	28 (22–45)	30 (21–48)	26 (22–39)	0.295
**Total serum bilirubin (mg/dl)**	0.6 (0.4–1)	0.8 (0.5–1)	0.6 (0.4–0.9)	0.196
**Serum albumin (g/dl) **	3.9 (3.3–4.2)	3.8 (3.4–4.1)	3.9 (3.3–4.4)	0.281
**Serum creatinine (mg/dl) **	0.9 (0.8–1.1)	1 (0.9–1.2)	0.9 (0.7–1)	0.067
**Serum uric acid (mg/dl) **	5.4 (4.4–6.5)	5.3 (4.4–6.2)	5.6 (4.6–6.5)	0.573

Bolded, italicized values represent significant *P* values < 0.05

In our study cohort, the MYD88 L265P mutation was noticed to be more frequent in younger patients (*p* = 0.022); however, no significant correlations were reached for the subtype or the anatomical site distribution.

**Fig. 2:**
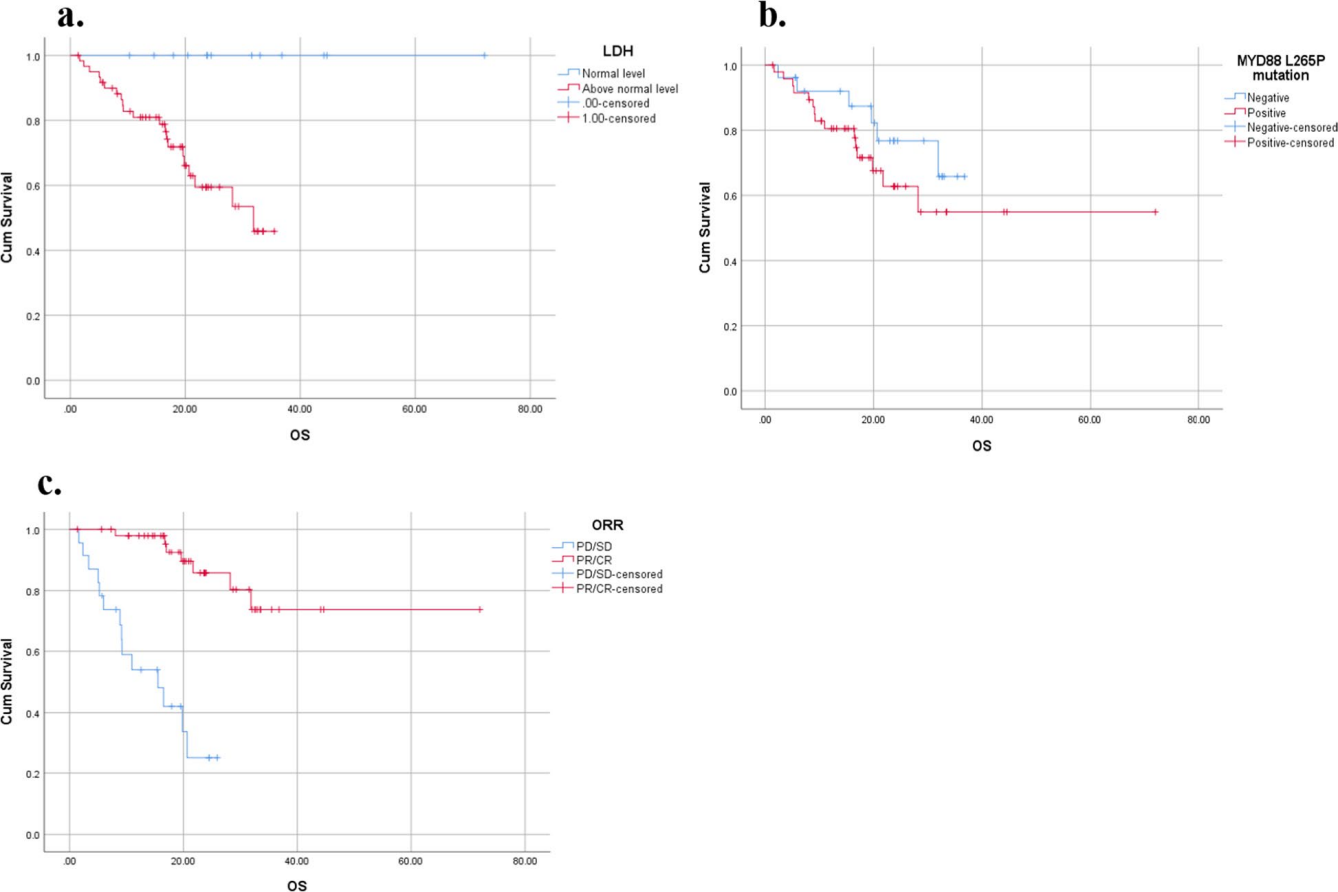
Kaplan–Meier survival curves based on **a** LDH level, **b** MYD88 L265 mutation, and **c** overall response rate (ORR) in DLBCL. The patients with high levels of LDH and patients with PD/SD (progressive disease/stable disease) show shortened OS, compared with the patients with low LDH levels and patients with PR/CR (partial response/complete response) (log-rank test, *p* = 0.008 and < 0.0001, respectively), while no significant difference was found in overall survival between MYD88 L265-mutated and non-mutated patients (log-rank test, *p* = 0.25)

**Fig. 3:**
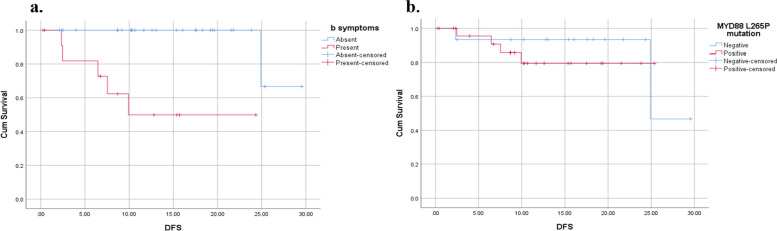
Kaplan–Meier survival curves based on **a** B symptoms and **b** MYD88 L265 mutation in DLBCL. The patients with B symptoms show shortened DFS (disease-free survival), compared with the patients without B symptoms (log-rank test, *p*** < **0.0001), while no significant difference was found in DFS between MYD88 L265-mutated and non-mutated patients (log-rank test, *p* = 0.54)

The MYD88 L265 mutation status did not impact treatment response or survival. Moreover, the ECOG performance of the MYD88-mutated patients was noticed to be slightly better. No significant associations with DFS and OS were found with the L265P mutation (*p* > 0.05) in all cases (Table 3, Figs. 2 and 3).

**Table 3:** Univariate analysis of overall survival and disease-free survival of DLBCL patients

**Parameters**	**Overall survival (OS)**		**Disease-free survival (DFS)**	***P* value**
	**Log rank**	***P* value**	**Log rank**	
**Age (≥ 60 vs < 60 years)**	0.81	0.37	1.22	0.27
**Gender (male vs female)**	3.9	***0.048***	1.6	0.21
**Performance status (< 2 vs ≥ 2)**	0.03	0.85	0.87	0.35
**HCV (seronegative vs seropositive)**	0	0.99	1.41	0.24
**B symptoms (absent vs present)**	0.04	0.83	14.98	***< 0.0001***
**LDH (normal vs > ULN)**	6.98	***0.008***	5.29	***0.02***
**IPI (low / low-intermediate vs high intermediate/ high)**	5.15	0.16	2.28	0.52
**CNS-IPI (low vs intermediate vs high)**	6.17	***0.046***	2.08	0.35
**Han’s Algorithm (ABC vs GC)**	0.75	0.38	2.45	0.12
**MYD88 L265P mutation (negative vs positive)**	1.28	0.25	0.54	0.54
**Overall response (CR/PR vs PD/SD)**	31.7	***< 0.0001***	-	-

Bolded, italicized values represent significant *P* values < 0.05

### MYD88 L265P mutation and survival analysis

The median follow-up time across the entire cohort was 26 months. Univariate analysis showed that overall survival (OS) was affected by gender, LDH level, and CNS-IPI scoring (*p* = 0.048,0.008, and 0.046, respectively), while disease-free survival (DFS) was affected by B symptoms and LDH level (*p* =  < 0.0001 and 0.02, respectively). However, the MYD88 mutation status and other prognostic factors showed no association with OS or DFS (Figs. 2 and 3 and Table 3).

## Discussion

Several prognostic mutations and many genetic alterations have been recently recognized in DLBCL. So, identifying the biological background of DLBCL patients with unfavorable outcome is of high clinical importance.

*MYD88* mutation was reported to activate both NF-κB and JAK/STAT signaling pathways [11]. Ngo et al. demonstrated that wild-type *MYD88* causes moderate activation of NF-κB, whereas L265P showed the strongest NF-κB activation. However, other isoforms showed much less capacity to activate NF-κB than L265P [11].

It was observed that the impact of *MYD88* mutations is variable among different lymphoproliferative disorders and cellular pathways affected and that *MYD88* mutations have different relevance for cell survival according to the stage of B-cell maturation. As noted, CLL patients with mutated *MYD88* show a favorable prognosis [23].

So, the identification and characterization of DLBCL patients with *MYD88* L265P mutation could provide a rationale for new modalities of target therapy. It has been recently investigated that DLBCL could be treated with L265P-derived peptide vaccination as a novel tumor-specific antigen to induce cytotoxic T-cell reaction [24].

Different techniques, such as Sanger sequencing and allele‑specific PCR (AS-PCR), have been used to detect MYD88 mutations [22]. Since Sanger sequencing might be unable to detect lower frequency mutations in FFPE samples with fragmented nucleic acids, AS-PCR was applied to detect the MYD88 L265P mutation, which is considered a highly sensitive and cost-effective technique [25].

In this study, we used AS-PCR for *MYD88* L265P mutation detection and identified high frequency in our DLBCL patients (59.8%), in contrast to previous studies that reported low *MYD88* L265P mutation frequency (6.5–29.6%) [11, 13, 26–30].

However, higher rates of MYD88 L265 mutation were previously detected using either digital droplet PCR where the L265 mutation was found in 29% of DLBCL patients [31], or with allele-specific semi-nested PCR (ASSN-PCR) observing *MYD88* L265P in 30.19% of all DLBCL patients treated with R-CHOP [32]. The variation in the frequencies of MYD88 L265P mutation among different studies might be attributed to the different ethnic and genetic backgrounds of the study groups and techniques used in mutation detection, in addition to the heterogeneity of the disease itself.

According to our results, *MYD88* L265P mutation was not found to significantly influence the clinicopathologic parameters of the DLBCL patients, similar to what was observed previously [28, 33, 34] demonstrating that the L265P mutation was not associated with clinicopathologic parameters of DLBC.

Significant association between the *MYD88* L265P mutation and old age was reported [16, 26, 28, 35]. However, our results indicated that the MYD88 L265P mutation was significantly associated with younger aged patients, with no association with gender or clinical stage.

Moreover, there was no significant association between *MYD88* L265P mutation and DLBCL subtype, similar findings were previously reported [16, 28]. However, Bohers et al. [13] demonstrated that *MYD88* L265P mutation was significantly higher in the ABC subtype, and even Ngo et al. [11] declared that the GCB subtype has almost no *MYD88* L265P mutation. The predominance of MYD88 L265P in non-GCB DLBCL was attributed to the frequent activation of NF-κB pathway in this subtype as explained by Kim et al. [28].

Previous studies demonstrated that the *MYD88* L265P mutation is markedly associated with lower survival rates [16, 26, 36], but this was not proved in a meta-analysis conducted by Lee et al. [10]; similarly, in our study, no relationship was found between the mutation and the overall survival.

It has been postulated [26] that patients with *MYD88* L265P showed poor PFS and OS due to other related variables such as older age or ABC subtype rather than the mutation itself, and the MyD88 L265P mutation itself does not have a significant effect on prognosis in systemic DLBCL [37].

Additionally, we found that the *MYD88* L265P mutation was not associated with treatment response or relapse; similar findings were reported by [33–35].

HCV core protein has been shown to be capable of directly interacting with TLR2 and resulting in activation of TLR2-MyD88 signaling cascade [38]. In our study, 47% of DLBCL patients were HCV seropositive at diagnosis. Interestingly, we found a significant association between MYD88 L265P mutation and seronegativity of HCV (*p* = 0.037), and this correlation was associated with significantly higher platelet count in MYD88 L265P-mutated patients.

This correlation needs further detailed study especially in our region where 72% of HCV-related lymphoma were previously observed in DLBCL patients [39].

Our study indicated that variables predicting poor overall survival and disease-free survival were gender, B symptoms, high serum LDH, and CNS-IPI scoring consistent with other reports [19, 32, 40] that showed an association of median LDH level and the presence of B symptoms with low OS and poor prognosis.

Limitations of our study include a relatively short follow-up period for survival analysis, and the sample size was relatively small. Some other limitations include non-homogeneous treatment of the enrolled patients, as the patient groups with or without rituximab treatment were combined. Therefore, further large-scale investigations using a more homogeneous population with a longer period of follow-up are warranted.

## Conclusion

Our study identifies that the *MYD88* L265P mutation is not significantly associated with the risk stratification of the disease, treatment response, or relapse and that *MYD88* L265P mutation may not serve as a prognostic marker for DLBCL patients in our locality. However, these data should be discussed in large-scale, multi-center, prospective studies with longer follow-up periods are recommended.

## Data Availability

All the data is available and shared in the manuscript.

## Abbreviations

DLBCL: Diffuse large B-cell lymphoma
MYD88: Myeloid differentiation primary response 88
OS: Overall survival
DFS: Disease-free survival
NHL: Non-Hodgkin’s lymphoma
COO: Cell of origin
GEP: Gene expression profiling
GCB: Germinal center B-cell like
ABC: Activated B-cell like
PMBL: Primary mediastinal B-cell lymphoma
BCR: B-cell receptor
TLR: Toll-like receptor
IRAKs: IL-1R-associated kinases
LDH: Lactate dehydrogenase
ECL: Electrochemiluminescence
FFPE: Formalin-fixed paraffin-embedded
PFS: Progression-free survival
IPI: International Prognostic Index
AS-PCR: Allele‑specific PCR
ASSN-PCR: Allele-specific semi-nested PCR
